# RNA Interference in Insects: Protecting Beneficials and Controlling Pests

**DOI:** 10.3389/fphys.2018.01912

**Published:** 2019-01-11

**Authors:** Elise Vogel, Dulce Santos, Lina Mingels, Thomas-Wolf Verdonckt, Jozef Vanden Broeck

**Affiliations:** Research Group of Molecular Developmental Physiology and Signal Transduction, KU Leuven, Leuven, Belgium

**Keywords:** insects, RNA interference, environmental RNAi, systemic RNAi, insecticides, delivery systems, viruses, antiviral immunity

## Abstract

Insects constitute the largest and most diverse group of animals on Earth with an equally diverse virome. The main antiviral immune system of these animals is the post-transcriptional gene-silencing mechanism known as RNA(i) interference. Furthermore, this process can be artificially triggered via delivery of gene-specific double-stranded RNA molecules, leading to specific endogenous gene silencing. This is called RNAi technology and has important applications in several fields. In this paper, we review RNAi mechanisms in insects as well as the potential of RNAi technology to contribute to species-specific insecticidal strategies. Regarding this aspect, we cover the range of strategies considered and investigated so far, as well as their limitations and the most promising approaches to overcome them. Additionally, we discuss patterns of viral infection, specifically persistent and acute insect viral infections. In the latter case, we focus on infections affecting economically relevant species. Within this scope, we review the use of insect-specific viruses as bio-insecticides. Last, we discuss RNAi-based strategies to protect beneficial insects from harmful viral infections and their potential practical application. As a whole, this manuscript stresses the impact of insect viruses and RNAi technology in human life, highlighting clear lines of investigation within an exciting and promising field of research.

## Introduction to Cell-Autonomous, Environmental and Systemic RNAi in Insects

The discovery of RNA interference (RNAi) constitutes an important milestone in the study of regulatory RNAs (Fire et al., 1998). In this process, small (s)RNA molecules of 18–31 nucleotides (nt) long effectuate a sequence-specific gene silencing response, acting at the post-transcriptional level through cleavage or blockage of longer RNAs containing a matching sequence (Siomi and Siomi, 2009). Based on their origin, biogenesis, structure and role in distinct biological processes, small RNAs are classified in three main cell-autonomous pathways: (1) genome encoded microRNAs (miRNAs), which regulate a multitude of biological processes; (2) PIWI-interacting (pi)RNAs, which silence transcripts derived from selfish genomic elements, such as transposons (Klattenhoff and Theurkauf, 2008); and (3) small interfering (si)RNAs, which defend the organism against invading viruses (Wang et al., 2006). However, recent studies revealed that some level of functional crosstalk can occur between the different sRNA-mediated pathways. A fascinating example is found in insects, where an important antiviral role of the piRNA pathway has been described in mosquitoes (Keene et al., 2004; Schnettler et al., 2013; Miesen et al., 2015, 2016; Palatini et al., 2017; Varjak et al., 2017).

In insects, the siRNA pathway is activated when double-stranded (ds)RNA molecules, as products of viral replication, are recognized in the cytoplasm and processed into siRNAs of 18-24 nt by the RNase type III enzyme Dicer-2 (Siomi and Siomi, 2009). Cleavage of viral RNA targets is then further exerted by an Argonaute-2 (Ago2) containing ‘RNA induced silencing complex’ (RISC), which encompasses the siRNA guide strand. Interestingly, this RNA silencing mechanism can also be triggered by artificial administration of gene-specific long dsRNA, a technique that is generally designated as RNAi (Wynant et al., 2014b). This dsRNA treatment can result in functional knockdown effects that in fact can be considered as auto-immune defects, since the siRNA pathway, an antiviral immune defense mechanism of insects, is being misled to target an endogenous transcript of the host. As such, RNAi has become the most widely used reverse genetics research tool in insects and holds great potential to contribute to novel strategies for species-specific control of insect pests and to combat viral infections in disease-vectoring and beneficial insects.

**FIGURE 1:**
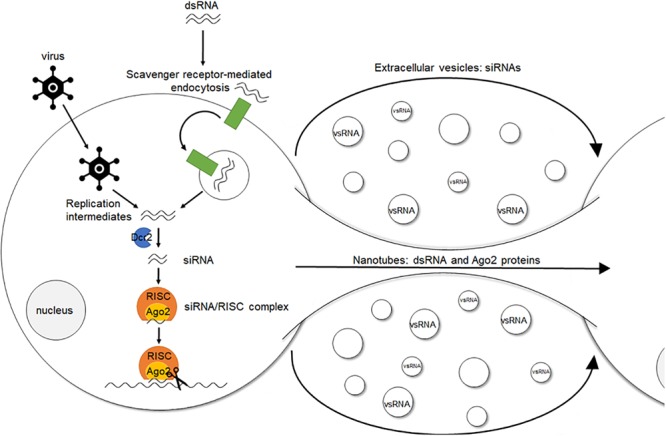
Simplified model of environmental, cell autonomous and systemic (antiviral) RNAi in insects. The siRNA pathway is triggered by dsRNA molecules. These duplexes naturally occur inside the cell during viral replication or can be artificially delivered. In the latter, cellular uptake of dsRNA, i.e., environmental RNAi, occurs via scavenger receptor-mediated endocytosis. Once inside the cell, i.e., cell autonomous RNAi, the dsRNA molecules are recognized in the cytoplasm and processed into siRNAs by Dcr2. Cleavage of viral RNA targets or endogenous transcripts is then further exerted by an Ago2-containing RISC, which encompasses the siRNA guide strand. Regarding antiviral RNAi in *Drosophila*, viral infection increases the formation of nanotube-like structures, through which short-distance transport of dsRNA and RISC components can occur. In addition, hemocyte-derived exosome-like vesicles systemically spread an antiviral RNAi signal (vsRNAs) in the hemolymph. This spread of the RNAi signal to cells in which the RNAi response had not been initiated before is named systemic RNAi. dsRNA, double stranded RNA. Dcr2, Dicer2. siRNA, small interfering RNA. RISC, RNA-induced silencing complex. Ago2, Argonaute2. vsRNA, viral small RNAs.

An interesting aspect of the RNAi response in insects is its potential systemic character, also known as systemic (sys)RNAi. Specifically, in some insects administration of dsRNA can result in the generation of an RNAi response throughout the entire body (Turner et al., 2006; Meyering-Vos and Müller, 2007; Bautista et al., 2009; Bolognesi et al., 2012; Wynant et al., 2012; Abd El Halim et al., 2016; Darrington et al., 2017). However, the precise mechanism of both short- and long-distance intercellular transfer of the sysRNAi-signal, as well as the exact nature of this signal, still remain elusive. Different reports indicate that the cellular uptake of dsRNA in insects, also referred to as environmental (env)RNAi, occurs via scavenger receptor-mediated endocytosis both in cultured cells and *in vivo* (Saleh et al., 2006; Wynant et al., 2014c). It is also known that the uptake of naked dsRNA is length-dependent. It occurs efficiently for long dsRNA molecules of around 200–500 base pairs (bp) and even of up to circa 1000 bp. However, for shorter constructs such as siRNAs, this efficiency decreases (Saleh et al., 2006; Huvenne and Smagghe, 2010; Bolognesi et al., 2012; Miller et al., 2012; Wang et al., 2013). Furthermore, it has been shown that lipophorins can adhere to dsRNA fragments in the insect hemolymph, suggesting a possible role of these proteins in either protection, transport, or both, throughout the body (Wynant et al., 2014a). In addition, two main findings have been reported for *Drosophila melanogaster*. First, viral infection of cultured *Drosophila* cells increased the formation of nanotube-like structures through which short-distance transport of dsRNA and RISC components can occur (Karlikow et al., 2016). Second, it has been shown that flies use hemocyte-derived exosome-like vesicles to systemically spread an antiviral siRNA signal in the hemolymph (Tassetto et al., 2017). At present, it is still unclear how all these separate findings might fit together and whether they can be extrapolated to other conditions and species (Figure 1).

In this review article, we start by discussing the potential of the RNAi technique to contribute to insect pest control. On this matter, we review several application strategies that have been tried, their limitations and the most promising approaches described in the available scientific literature thus far. On a parallel perspective, we summarize relevant insect viral infections and review the use of viruses as bio-insecticides. Finally, with perspective to the natural antiviral role of the RNAi mechanism, we discuss the potential use of RNAi for protecting beneficial insects from harmful viral diseases.

## RNAi-Based Insect Pest Control

Despite frequent use of insecticides, approximately 18–20% of the global crop harvest is still lost due to damage caused by pest insects (Sharma et al., 2017). A major underlying cause is insect population resistance against the most commonly used insecticides, posing a persistent challenge to agriculture (Tabashnik et al., 2013; Zhu et al., 2016). Furthermore, the devastating impact of chemical insecticides on the environment and other organisms, such as beneficial insects, can no longer be ignored (Ansari et al., 2014). Taking the previous statements into account, it becomes evident that the current array of insect pest combatting methods is insufficient to secure global food production for the next decades. Finding alternative options to improve plant protection strategies is therefore critical.

In this context, an interesting perspective is represented by the RNAi technique. The potential of this mechanism is inherent in its mode-of-action, namely the subsequent degradation of complementary target mRNA upon entry of specific dsRNA into the cell (Agrawal et al., 2003). Therefore, by delivering dsRNA targeting any endogenous gene transcript to the intended pest organism, expression of this gene can be knocked down at the post-transcriptional level. Thus, through careful selection of an essential target gene, this mechanism can lead to insect mortality. The sequence-specific nature and the possibility to theoretically target any non-conserved, ‘lethal’ gene, make RNAi an ideal candidate for further application as a species-specific insecticide.

A proof-of-concept study was executed in 2007 by Baum et al. In this research, a transgenic corn crop was genetically engineered to express dsRNA against the V-ATPase A transcript of the Western corn rootworm *Diabrotica virgifera virgifera*. Feeding *D. virgifera virgifera* with this modified plant resulted in larval stunting and in the premature death of the insect. Additionally, dsRNA functioned as a crop protectant as feeding damage to the transgenic corn was greatly reduced (Baum et al., 2007). A similar study was executed for the cotton bollworm *Helicoverpa armigera*. In this research, Mao et al. (2007) showed that plant-mediated expression of dsRNA targeting the cytochrome P450 monooxygenase gene (CYP6AE14) could increase the toxic effects of gossypol, a cotton metabolite that is otherwise tolerated by the cotton bollworm. Silencing of CYP6AE14 led to delayed larval growth when gossypol was supplemented in the diet (Mao et al., 2007). It should be noted that another research has since shown that CYP6AE14 is likely not directly involved in gossypol metabolism but rather plays a more general role in the insect stress response to ingestion of plant toxins (Krempl et al., 2016). Nonetheless, the research of Mao et al. remains an interesting example of the application potential of dsRNA-mediated plant protection.

This section will continue by reviewing the predominant theories regarding the variable nature of the RNAi response across the class Insecta. Bearing in mind the promising use of RNAi technology as an insecticide, an overview of dsRNA delivery systems is given next. Finally, current use of RNAi-based insecticides will be summarized.

### Variable Efficiency of the RNAi Response

Although an RNAi response has been detected at least once in most economically important insect orders, such as Coleoptera, Diptera, Orthoptera, Lepidoptera, and Hemiptera, the efficiency of the induced response may vary between species and even within the same organism (Bellés, 2010; Wynant et al., 2014b; Xu et al., 2016). Whereas some insects, such as the Colorado potato beetle *Leptinotarsa decemlineata*, and the western corn rootworm *D. virgifera virgifera*, are consistently able to generate a systemic RNAi response; other species, such as the tobacco cutworm *Spodoptera litura*, and the silkworm *Bombyx mori*, show a more variable and generally less efficient response (Terenius et al., 2011). The nature of this variable efficiency has been the subject of much speculation and it is likely that a lot remains to be discovered.

#### Extracellular Nucleases Inhibit the Efficiency of the RNAi Response

Some insect species, such as the desert locust *Schistocerca gregaria*, and the migratory locust *Locusta migratoria*, are able to display an efficient systemic RNAi response after injection of dsRNA into the body cavity (Luo et al., 2012; Wynant et al., 2012). However, when dsRNA is fed to these insects, they appear to be refractory (Luo et al., 2013; Wynant et al., 2014c). As most food sources contain nucleic acids, it follows that nucleases are an integral part of the insect digestive system. In addition, it has been suggested that nucleases in the digestive track may also exert a function of protection against viruses (Musser et al., 2002). Unfortunately, presence of high nuclease activity can have an inhibitory effect on the RNAi response (Katoch and Thakur, 2012).

Wynant et al. (2014d) showed that a digestive enzyme solution, collected from the midgut of *S. gregaria*, had the ability to quickly degrade dsRNA (i.e., 150 nanograms of dsRNA within 5 min). Subsequently, four different sequences coding for dsRNases were identified from *S. gregaria* transcriptome data (Wynant et al., 2014d). In several other species, such as the pea aphid *Acyrthosiphon pisum*, the tarnished plant bug *Lygus lineolaris, B. mori*, and *L. migratoria*, the limited RNAi response after feeding with dsRNA has been linked to the presence of dsRNA-degrading enzymes in the digestive system (Arimatsu et al., 2007; Allen and Walker, 2012; Liu et al., 2012; Luo et al., 2013; Christiaens et al., 2014).

The limiting effect of nucleases is not unique to the digestive tract. In fact, their activity in the hemolymph has been linked to a lowered RNAi efficiency in a number of species (Singh et al., 2017). Whereas dsRNA remained stable in the hemolymph of the RNAi efficient German cockroach *Blattella germanica*, injection of dsRNA into the body cavity of the RNAi refractory tobacco hornworm *Manduca sexta*, led to its swift degradation (Garbutt et al., 2013). Furthermore, in the Asian corn borer *Ostrinia furnacalis*, the nuclease Rease was found to be upregulated in the hemolymph after administration of dsRNA (Guan et al., 2018). Reducing transcript levels of this gene led to a significantly improved RNAi response in this insect. Phylogenetic analysis revealed homologous genes in seven other lepidopteran species, suggesting that this nuclease might be Lepidoptera-specific (Singh et al., 2017).

Surprisingly, the presence of dsRNases has even been recorded in insects which generally show an efficient RNAi response after feeding with dsRNA, such as the Colorado potato beetle *L. decemlineata* (Singh et al., 2017; Spit et al., 2017). Spit et al. (2017) showed that an enzyme solution collected from the gut of this beetle was capable of degrading dsRNA. Additionally, the induced RNAi response after feeding dsRNA could still be significantly increased after the knockdown of two dsRNA degrading enzymes, Ld_dsRNase1 and Ld_dsRNase2 (Spit et al., 2017). Although it has become clear that most insect orders contain species wherein the efficiency of the RNAi response is somehow limited by the presence of dsRNases, not all species appear to be equally affected. It is therefore likely that differences in the activity of dsRNA degrading enzymes contribute to the tissue-, stage- and species-dependent variability in RNAi sensitivity observed in insects (Singh et al., 2017). In this context, it is notable that extracellular nuclease activity will additionally influence dose-dependence of the RNAi response as it conditions the quantity of dsRNA that remains available for uptake. Certainly, it appears that the role of dsRNases in limiting the RNAi response cannot be ignored. Moreover, since reducing transcript levels of dsRNases already led to an improved RNAi sensitivity in some insect species, these nucleases may have to be taken into account when considering future RNAi-based insect pest control strategies (Guan et al., 2018).

#### Tissue-Dependence of the RNAi Response

The fruit fly *D. melanogaster*, is a well-known example of an insect that is recalcitrant to external administration of dsRNA (Whyard et al., 2009). Remarkably, this is a generalization that does not apply to the whole insect: *D. melanogaster* hemocytes can take up extracellular dsRNA and generate an RNAi response. This sensitivity can also be observed in the hemocyte-derived *D. melanogaster* S2 cell line, a commonly used *in vitro* model for RNAi research (Clemens et al., 2000).

While this inconsistency is striking, the fruit fly is not the only organism in which the sensitivity of the RNAi response appears to be cell type or tissue-dependent. Another striking example presents itself in *S. gregaria*; independent studies in this species have proven that an efficient RNAi response can be induced in various tissues, ranging from the brain to the Malpighian tubules (Badisco et al., 2011; Marchal et al., 2011a,b, 2012; Ott et al., 2012; Van Wielendaele et al., 2012; Lenaerts et al., 2016, 2017a,b). Nevertheless, Wynant et al. (2012) have observed that the ovaries and testes of this locust species showed a lower RNAi efficiency when compared to these other tissues. Further examples can be found in the African malaria mosquito *Anopheles gambiae*, and the yellow fever mosquito *Aedes aegypti*. In the former, a reduced RNAi susceptibility was observed in the salivary glands, whereas in the latter both head and ovarian tissues responded less efficiently than other tissues to external application of dsRNA (Boisson et al., 2006; Telang et al., 2013). Finally, lepidopterans are known to have a variable RNAi susceptibility and tissue-dependency has also been observed for a number of species in this order. Particularly wing-disk and larval epidermal tissues appear to be problematic (Terenius et al., 2011).

Little is known about the exact causes of the tissue-dependency of the RNAi response, and as such they may vary between and within species. Research using the lepidopteran Sf9 and Hv-E6 cell lines showed that dsRNA molecules are unable to escape after endosomal uptake (Shukla et al., 2016). Thus, in these insect cells, the RNAi response is not induced because the dsRNA cannot enter the cytoplasm. In this context, it has been suggested that this inability might contribute to the poor susceptibility and possibly to the tissue-dependency often observed in this insect order (Shukla et al., 2016). Similarly, in *L. migratoria* it was found that a reduced uptake of dsRNA in oocytes and follicle cells resulted in a less efficient ovarian RNAi response (Ren et al., 2014). In *S. gregaria*, the reduced responsiveness of ovaries and testes could be attributed to reduced expression levels of *argonaute-2* and *dicer-2*, two crucial RNAi genes (Wynant et al., 2012). Likewise, in several lepidopteran species it was also suggested that variable expression of core RNAi components might be linked to the inconsistent RNAi response observed in these insects (Terenius et al., 2011; Garbutt and Reynolds, 2012).

#### Intra-Species Differential Sensitivity and Resistance

To fully understand the problem of variable RNAi efficiency in insects, intraspecies variations also need to be considered. Indeed, some populations of the same species appear to be differentially sensitive to external administration of dsRNA. This was most recently observed in *L. migratoria* by Sugahara et al. (2017). In this research, four different lab strains of the migratory locust were examined, each originating from a geographically isolated location in Japan. Two of the tested lab strains were found to be very sensitive to injection with dsRNA, while the other two appeared to be completely refractory. Even within the same lab strain, different individuals could respond with different degrees of sensitivity (Sugahara et al., 2017). In a comparable research, three phenotypically different field populations of *D. virgifera virgifera* were given the same dsRNA treatment to see whether they would respond in a similar way. The efficiency of the RNAi treatment varied between the three populations, indicating that the RNAi susceptibility differed for each population (Chu et al., 2014). It has been suggested that a similar phenomenon could be occurring in the red flour beetle, *Tribolium castaneum* (Spit et al., 2017). While some lab strains of this species show a highly sensitive response to feeding with dsRNA, other lab strains appear to be unresponsive to this method of administration (Whyard et al., 2009; Miyata et al., 2014; Abd El Halim et al., 2016; Spit et al., 2017).

The cause for these intraspecies variations in RNAi sensitivity remains uncertain. Sugahara et al. (2017) proposed that in locusts these intraspecies differences could be attributed to a genetic component. They postulate that RNAi sensitivity is regulated by an incompletely dominant gene or several genes that remain to be determined (Sugahara et al., 2017). On the other hand, another theory suggests persistent viral infections as a key-determining factor in the establishment of variable RNAi efficiencies between insect populations of the same or different species. Swevers et al. (2013) hypothesize that these infections could reduce insect RNAi sensitivity through the expression of viral suppressors of RNAi, the saturation of the RNAi machinery by viral siRNAs, or the manipulation of host gene expression. However, this remains speculative as the effect of specific persistent viral infections on the efficiency of the RNAi response in lepidopteran cells remains to be demonstrated (Swevers et al., 2016). Thus, further investigation is necessary to verify this hypothesis.

Taken together, observations regarding the variation of RNAi efficiency suggest that the potential emergence of RNAi-resistance in currently RNAi-sensitive insects is a real possibility. Indeed, in a recent study, an RNAi-resistant insect population was created. Resistance was induced by consistently feeding a field population of the Western corn rootworm with transgenic maize plants expressing *DvSnf7* dsRNA. Khajuria et al. (2018) showed that resistant insects displayed reduced dsRNA uptake from the gut lumen after feeding. Furthermore, the researchers were able to determine that the resistance was not limited to *DvSnf7* dsRNA, since the insects displayed cross-resistance to feeding with several different dsRNAs (Khajuria et al., 2018). Further research into the underlying mechanisms causing the development of resistance in RNAi-sensitive insects could provide important insights into the efficient application of RNAi as an insecticide.

#### Target Selection and Construct Design

Taking the aforementioned variabilities into account, it is unsurprising that the set-up of RNAi technology experiments requires careful consideration. Selection of the target gene of interest, for instance, is crucial to their success or failure. The ideal target gene should be abundantly transcribed, produce an mRNA with a high turnover rate and translate into a protein with a low half-life (Scott et al., 2013). To become applicable as an insecticide, transcript reduction of the intended target gene must additionally lead to mortality in the insect. Furthermore, off-target effects should always be considered. With regard to this, dsRNA constructs should preferably be chosen in non-conserved regions of the target mRNA to avoid cross-silencing among other species or isoforms of the gene of interest. Correspondingly, research has shown that RNAi can be highly species-specific if the dsRNA construct is well-designed. By targeting the variable 3′-UTR region of the greatly conserved γ-Tubulin transcript, Whyard et al. (2009) showed that a species-specific knockdown could even be achieved in four closely related *Drosophila* species. Similarly, Kumar et al. (2012) could induce very specific transcript reductions for three highly similar CYP genes in the Tobacco hornworm *Manduca sexta*.

Moreover, the length of the dsRNA construct should be contemplated as the optimal length for dsRNA uptake varies from insect to insect (Bolognesi et al., 2012). Research has shown that for most insects this optimum lies between 200 and 520 bp (reviewed by Huvenne and Smagghe, 2010). A last criterium that should be taken into account is the dosage of dsRNA that is administrated. This concentration should be adjusted according to the abundance of target mRNA. As most genes are not stably expressed during the entire life cycle of the insect, temporal expression according to life and developmental stage should be taken into account. In addition, this concentration can be species dependent. In insect species where dsRNAses limit RNAi efficiency in the gut, for instance, an overdose of dsRNA may be required to induce an RNAi signal. This was extensively reviewed by Scott et al. (2013).

### RNAi Delivery Systems

Clearly the obstacles of insufficient RNAi sensitivity must be solved before RNAi technology can be further applied as a universal insecticide. An elegant solution is presented by packaging dsRNA in such a way that it is protected against degradation and uptake is facilitated. This may be achieved through the use of delivery systems. Many different strategies have already been proposed in the existing literature and will be discussed here. An important feat to keep in mind is that the specificity of these systems has to be prudently considered. As such, the effect of all proposed delivery strategies on other animals and on human consumption has to be investigated thoroughly before they can be applied as vehicles for insecticidal dsRNA. Regardless, the great potential of these systems is undeniable. An overview of all delivery systems reported so far can be found in Table 1.

**Table 1:** Overview of delivery systems used for the successful delivery of dsRNA in several economically important insect orders.

Insect order	Category	Delivery system	Species	Target gene^∗^	Reference
Lepidoptera	Micro-organism	Bacteria	*Spodoptera exigua*	*Chitin synthase A (SeCHSA)*	Tian et al., 2009
			*Spodoptera exigua*	*Chymotrypsin 2 (SeCHY2)*	Vatanparast and Kim, 2017
			*Helicoverpa armigera*	*Ultraspiracle protein (USP)*	Yang and Han, 2014
			*Sesamia nonagrioides*	*Juvenile hormone esterase (SnJHE)*	Kontogiannatos et al., 2013
	Viral	BmNPV	*Sesamia nonagrioides*	*Juvenile hormone esterase (SnJHE)*	Kontogiannatos et al., 2013
		AcMNPV	*Heliothis virescens*	*Juvenile hormone esterase (HvJHE)*	Hajós et al., 1999
		Sindbis Virus	*Bombyx mori*	*Broad-Complex (Br-C)*	Uhlirova et al., 2003
	Nanoparticle	FNP	*Ostrinia furnacalis*	*Chitinase-like gene CHT10*	He et al., 2013
		Guanylated polymers	*Spodoptera exigua*	*Chitin synthase B*	Christiaens et al., 2018
Coleoptera	Micro-organism	Bacteria	*Leptinotarsa decemlineata*	*β-actin (actin), Protein transport protein sec23 (Sec23), Coatomer subunit beta (COPβ)*	Zhu et al., 2011
	Proteinaceous	PTD-DRBD	*Anthonomus grandis*	*Chitin synthase II* (*AgChSII*)	Gillet et al., 2017
Hemiptera	Micro-organism	Bacterial symbiont – *Rhodococcus rhodnii*	*Rhodnius prolixus*	*Nitrophin 1 (NP1), Nitrophin 2 (NP2), Vitellogenin (Vg)*	Whitten et al., 2016
		Bacterial symbiont – *BFo2*	*Frankliniella occidentalis*	*α-Tubulin (Tub)*	Whitten et al., 2016
Diptera	Micro-organism	Yeast symbiont – *Saccharomyces cerevisiae*	*Drosophila suzukii*	*γ-Tubulin 23C (γTub23C))*	Murphy et al., 2016
		*Chlamydomonas reinhardtii*	*Anopheles stephensi*	*3-hydroxykynurenine transaminase (3-HKT)*	Kumar et al., 2013
		*Pichia pastoris*	*Aedes aegypti*	*Juvenile hormone acid methyl transferase* (*AeaJHAMT*)	Van Ekert et al., 2014
	Nanoparticles	*Chitosan*	*Aedes aegypti*	*Semaphorin-1a (sema1a)*	Mysore et al., 2013
			*Aedes aegypti*	*Single-minded (Sim)*	Mysore et al., 2014
			*Aedes aegypti*	*Vestigial gene (vg)*	Kumar et al., 2016
			*Anopheles gambiae*	*Chitin synthase 1 (AgCHS1), Chitin synthase 2 (AgCHS2)*	Zhang et al., 2010
	Liposomes	Lipofectamine 2000, Cellfectin, Transfectin, BMRIE-C	*Drosophila melanogaster*	*γ-Tubulin (γ-Tub)*	Whyard et al., 2009
			*Drosophila sechellia*	*γ-Tubulin (γ-Tub)*	Whyard et al., 2009
			*Drosophila yakuba*	*γ-Tubulin (γ-Tub)*	Whyard et al., 2009
			*Drosophila pseudoobscura*	*γ-Tubulin (γ-Tub)*	Whyard et al., 2009
		Lipofectamine 2000	*Drosophila suzukii*	*Alpha-coatomer protein (alpha COP), Ribosomal protein S13 (RPS13), Vacuolar H[+]-ATPase E subunit (Vha26)*	Taning et al., 2016
		Effectene	*Aedes aegypti*	*Inositol-requiring enzyme 1 (Ire-1), X-box binding protein-1 (Xbp-1), Caspase-1 (Cas-1), SREBP cleavage-activating protein (Scap), site-2 protease (S2P)*	Bedoya-Pérez et al., 2013
			*Aedes aegypti*	*Mitogen-activated protein kinase p38*	Cancino-Rodezno et al., 2010

*^∗^All target genes listed gave a more efficient knockdown than similar treatment with naked dsRNA*.

#### Micro-Organisms

The bacterial system, in its simplicity, is one of the most successful methods of dsRNA delivery in insects. This system makes use of the genetically modified HT115 bacterial strain, which lacks the dsRNA-degrading bacterial endonuclease RNase III. Furthermore, this strain contains the T7 polymerase gene, controlled by the inducible lac operon. HT115 is often combined with L4440, a plasmid specifically designed to contain two T7 promoters flanking its multiple cloning site. Transformation of the bacteria with L4440 will lead to expression of dsRNA within the cell. This method was first utilized in the nematode *Caenorhabditis elegans* (Timmons and Fire, 1998; Timmons et al., 2001). Since then it has also been applied to insects, as described below.

Tian et al. (2009) first fed bacteria expressing *Chitin synthase A* (*SeChsA*) dsRNA to the beet armyworm *Spodoptera exigua* in 2009. They found that this delivery method not only lead to an efficient knockdown but also to reduced larval growth and insect death (Tian et al., 2009). Moreover, feeding dsRNA in this way induced sysRNAi as reduced transcript levels were observed in the trachea and epidermis of treated insects (Tian et al., 2009). A similar experiment was performed in *L. decemlineata*, where a knockdown was achieved for several target genes after the insects were fed with dsRNA-expressing bacteria. In addition, the insects showed increased mortality as well as reduced weight gain (Zhu et al., 2011). Finally, bacterial delivery of dsRNA targeting the *ultraspiracle* gene transcript was shown to improve the RNAi efficiency through feeding in *H. armigera* (Yang and Han, 2014).

The mechanism through which this bacterial system facilitates dsRNA-uptake remains elusive. It is likely, however, that packaging dsRNA in a protective bacterial shell may have a stabilizing effect on the presence of dsRNA in the lumen of the digestive system. With regard to this, pre-treatment of the bacteria was shown to improve release of dsRNA in insects. Specifically, research has shown that sonication improved the efficiency of the induced RNAi response in *S. exigua* (Vatanparast and Kim, 2017). Therefore, it is possible that in this case weakening the bacterial cell wall through pretreatment stimulated dsRNA-uptake (Vatanparast and Kim, 2017). However, there is no concrete evidence for this and it remains to be proven.

The potential pathogenicity of *Escherichia coli* to several insect species implies that beneficial insects could be negatively affected by the use of this delivery system. Therefore, it has been suggested that a more appropriate approach might be achieved by focusing on symbiotic bacteria or yeasts. In the bloodsucking insect *Rhodnius prolixus*, and the western flower thrips *Frankliniella occidentalis*, knockdowns were achieved by delivering genetically engineered symbiotic bacteria capable of expressing dsRNA (Whitten et al., 2016). Furthermore, research suggests that this method of feeding might potentially lead to horizontal transfer of the RNAi signal in *R. prolixus* through symbiont-contaminated feces. More specifically, the eGFP-tagged symbiont could be detected in untreated younger instar insects after they had been fed with feces from treated insects. This implies that, through the use of this symbiont, possibly whole colonies could be targeted with only a minimal amount of bacteria (Whitten et al., 2016). Likewise, in the spotted wing fruit fly, *Drosophila suzukii*, it was found that feeding with a genetically modified symbiotic yeast led to induction of the RNAi response and resulted in reduced larval fitness (Murphy et al., 2016). Finally, it was shown that a knockdown could be achieved by feeding larvae of the mosquito species *Anopheles stephensi*, with a dsRNA delivery system consisting of transgenic microalgae (Kumar et al., 2013). These alternative delivery vehicles were suggested to have a negligible pathogenic impact on non-target insects, making them attractive options for application as ecologically friendly insecticides in the field.

#### Viruses

Viruses are extremely efficient at infecting cells and thus at delivering nucleic acid material into the intracellular environment. As RNAi is known to play a vital part in insect antiviral immunity (Bronkhorst and Van Rij, 2014), the use of viral delivery systems becomes an intriguing pitch, since the natural path of dsRNA cell entry is simulated. Furthermore, as many viruses have a very specific host range, a high degree of species-specificity could be achieved through careful virus screening and selection (Kolliopoulou et al., 2017). However, despite its many positive facets, viral delivery of dsRNA is still faced with a number of obstacles. Since many viruses have developed counter-measures against the RNAi mechanism, such as viral suppressors of RNAi, it is likely that not all viruses will be equally applicable as a delivery system (Swevers et al., 2013; Kolliopoulou et al., 2017). Some examples of successful experimental use of a viral delivery system are given below.

Kontogiannatos et al. (2013) found that a recombinant BmNPV baculovirus, encoding a juvenile hormone esterase specific hairpin, could induce gene-specific knockdown phenotypic effects in the Mediterranean corn borer, *Sesamia nonagrioides*. Surprisingly, despite careful selection of the viral carrier, the virus itself also seemed to affect the vitality of the insect. Therefore, it is likely that not all observed phenotypic effects could be attributed to the knockdown (Kontogiannatos et al., 2013). In *B. mori*, it was discovered that injection with a recombinant Sindbis virus (SINV) could achieve a knockdown of the transcription factor Broad-Complex (Br-C). Uhlirova et al. (2003) determined that engineering SINV to express an antisense RNA strand for Br-C led to reduced Br-C mRNA levels in this insect. This resulted in decreased rates of larval to pupal molting as well as developmental defects in those larvae that were able to reach adulthood (Uhlirova et al., 2003). It is of interest to mention that recombinant strains of SINV have additionally been used as a viral delivery system in the mosquito, *Aedes aegypti*, as a control measure for the RNAi-induced inhibition of dengue virus (Adelman et al., 2001).

While viral delivery systems show a lot of potential and are generally considered to be among the most efficient methods for dsRNA delivery, their *in vivo* application has not been widely investigated yet (Kolliopoulou et al., 2017). This may be due to the many safety issues that accompany this method of delivery. As not all insect viruses have a specific host range, a biosafety issue that needs to be thoroughly considered is cross-infection of beneficial insects with these highly virulent delivery systems. Furthermore, the ecological implications of releasing transgenic viruses into the field will need to be carefully considered, especially with regard to stability and turn-over time. A last point that will need to be evaluated is the possibility of transgene transfer from recombinant viral vesicles to wild type viruses (Kolliopoulou et al., 2017).

#### Nanoparticles

In order to increase stability and uptake efficiency, dsRNA can also be incorporated into a nanoparticle. Nanoparticles are polyplex-based delivery systems, consisting of either natural or synthetic polymer subunits. The most utilized nanoparticles are chitosan-derived.

Chitosan is a non-toxic, biodegradable molecule that can be obtained by deacetylation of chitin, one of the most abundant biopolymers in nature that is especially known for its structural function in the exoskeleton of arthropods (Dass and Choong, 2008). Due to its poly-cationic character and many amino groups, chitosan is able to bind dsRNA through electrostatic interaction. Chitosan:dsRNA nanoparticles are thus formed through self-assembly during the binding process. Incorporation of dsRNA into such a nanoparticle complex increases stability and uptake of the dsRNA *in vivo* (Zhang et al., 2010). This method of oral dsRNA delivery appeared to be especially effective in the African malaria mosquito *A. gambiae*, and the yellow fever mosquito *A. aegypti*. In these two species successful application of chitosan-mediated dsRNA delivery led to a knockdown in various independent experiments (Zhang et al., 2010; Kumar et al., 2013; Mysore et al., 2013, 2014; Zhang X. et al., 2015).

Additionally, nanoparticles can consist of synthetically modified polymers. An interesting example is presented by He et al. (2013), who generated a fluorescent nanoparticle (FNP) to facilitate dsRNA uptake in the Asian corn borer *Ostrinia furnacalis*. FNP consists of a core chromophore, allowing FNP uptake to be observed through fluorescence microscopy, and two outer shell layers that facilitate binding to dsRNA and prevent aggregation in water (He et al., 2013). Complexation of FNP with dsRNA targeting the chitinase-like gene CHT10 caused RNAi silencing after feeding of the Asian corn borer larvae. The treatment resulted in molting defects, reduced larval weight and, eventually, death (He et al., 2013). Finally, Christiaens et al. (2018) developed a series of nanoparticles designed to specifically shield dsRNA from the degrading effects of the highly basic conditions (high pH) that are typical of the lepidopteran gut. In this research, nanoparticle stability in this alkaline environment was enhanced by modifying cationic polymethacrylate derivatives with protective guanidine side groups. Feeding larvae of the beet armyworm S. *exigua* with *chitin synthase B* dsRNA packaged in these pH-stable nanoparticles, led to the swift knockdown of the target gene as well as increased mortality in the experimental insects (Christiaens et al., 2018).

#### Liposomes

Another means to obtain an increased RNAi efficiency is through the use of lipid-based transfection agents; these vesicles are collectively referred to as liposomes. Liposomes form naturally when transfection agents are brought into an aqueous environment. During this process, the positively charged lipids envelop the negatively charged nucleic acid material, forming compact lipid bilayer particles similar to the phospholipid bilayer of the cell membrane (Dalby et al., 2004). Cell entry of the liposome-encapsulated dsRNA is then achieved through lipofection.

Whyard et al. (2009) first used liposomes to improve the RNAi efficiency in four distinctive drosophilid species: *D. melanogaster, D. sechellia, D. yakuba*, and *D. pseudoobscura*. By creating liposomes using commercial transfection agents, such as Lipofectamine 2000 and Cellfectin (both available at Invitrogen), the dsRNA-induced mortality was increased (Whyard et al., 2009). Furthermore, mRNA silencing could be improved in the mosquito species *A. aegypti* by feeding it dsRNA packaged in Effectene-liposomes (Cancino-Rodezno et al., 2010; Bedoya-Pérez et al., 2013). Notably, a similar approach also led to liposome-mediated uptake of dsRNA in the tick species *Rhipicephalus haemaphysaloides* (Zhang et al., 2018).

#### Proteinaceous Delivery Systems

The use of carrier proteins as delivery systems for dsRNA also provides an interesting prospect. Although research within this category remains limited, the best studied protein carriers are represented by the so-called cell-penetrating peptides or CPPs. One of the characterizing traits of these peptides is that they are able to facilitate entry into the intracellular environment while transporting molecular cargo, such as dsRNA. CPPs are short chain cationic peptides that usually consist of 10 – 30 amino acids with a high prevalence of basic residues, such as lysine and arginine (Durzyńska et al., 2015). Although there is still some speculation about the exact cellular mechanisms of CPP-mediated delivery, the most commonly accepted theory is that endocytosis plays an important part (Choi and David, 2014).

To induce an RNAi response through feeding in the Cotton boll weevil *Anthonomus grandis*, a fusion protein was designed containing a peptide transduction domain (PTD) as well as the dsRNA binding domain (DRBD) from the human protein kinase R (Gillet et al., 2017). PTD is an enhanced version of the arginine-rich CPP *trans*-activating transcriptional activator (TAT) of the human Immunodeficiency Virus 1 (HIV-1), engineered to have additional properties that promote endosomal escape of the fusion protein and its cargo into the cytoplasm (Vivès et al., 1997; Wadia et al., 2004). PTD-DRBD, in combination with dsRNA, forms a ribonucleoprotein particle (RNP) that is able to swiftly facilitate uptake in the insect gut. Furthermore, after feeding RNPs to *A. grandis*, Gillet et al. (2017) found that the knockdown for *chitin synthase II* was significantly increased compared to feeding with naked dsRNA.

The CPPs may represent an intriguing solution to the problem of RNAi sensitivity. This category of compounds encompasses a wide diversity of untested candidates and, therefore, many potentially interesting carriers for oral dsRNA delivery remain to be discovered. However, some caution must be exercised as these CPPs belong to a class of very general protein carriers, able of entering mammalian cells as well as arthropod cells (Fawell et al., 1994; Vivès et al., 1997; Wadia et al., 2004; Durzyńska et al., 2015).

#### Chemical Modifications of Small RNA Oligonucleotides

Although the RNAi response is not effective upon exposure to short dsRNA duplexes such as siRNAs, it is known that chemical modifications of these molecules can improve their stability and uptake (Joga et al., 2016). In fact, feeding of modified siRNAs targeting vital genes can lead to mortality in the diamondback moth, *Plutella xylostella* (Gong et al., 2011, 2013). Furthermore, the use of modified synthetic miRNA inhibitors, antagomirs and agomirs is an interesting approach that requires further investigation (Liu et al., 2014; Li X. et al., 2015; He et al., 2017).

#### Plastids: A Plant-Based Delivery System

dsRNA delivery through genetically engineered plants has been achieved for many insect species, often resulting in reduced growth and developmental delay (Baum et al., 2007; Mao et al., 2007; Pitino et al., 2011; Kumar et al., 2012). Since plants possess their own RNAi machinery, transgenic dsRNAs produced *in planta* are swiftly diced into siRNAs instead of accumulating (Vazquez et al., 2010). However, efficient uptake of dsRNA in insects requires that administrated duplexes have a minimum length of 60 bp (Bolognesi et al., 2012). Therefore, as insects take up siRNAs much less efficiently than long dsRNAs, the corresponding toxicity of the transgenic plant will also be reduced.

While it is debatable whether plants really can be classified as typical delivery systems, an interesting cross-over presents itself in the research of Zhang J. et al. (2015). In their research, a potato plant was genetically engineered to produce dsRNAs in chloroplasts, a plant organelle that lacks the RNAi pathway thus allowing long dsRNAs to accumulate here. The dsRNA molecules expressed in these transgenic plants targeted β*-actin* and *Shrub.* Feeding larvae of the Colorado potato beetle with leaves from this modified potato plant resulted in 100% RNAi-induced mortality (Zhang J. et al., 2015). Thus, the chloroplasts function as a kind of delivery system within the plant, ensuring that dsRNA of the correct length reaches the target insect. Naturally, this discovery has major implications for the further mode of application of RNAi insecticides in the field.

### Current RNAi-Based Insecticides

The RNAi response has been thoroughly researched in the Western corn rootworm (WCR) *D. virgifera virgifera* (Baum et al., 2007; Rangasamy and Siegfried, 2012; Wu et al., 2017, 2018; Camargo et al., 2018). The WCR is a well-known pest insect with a significant economic impact on the maize harvest in the United States, as well as in Europe. In fact, it is estimated that in the United States alone, crop losses due to this plague amount to more than $1 billion annually (Sappington et al., 2006). The WCR has a very sensitive RNAi response to oral administration of dsRNA and many target genes with lethal or detrimental effects have already been identified in this insect (Baum et al., 2007). It is therefore not so surprising that the first RNAi-based insecticides for the control of this insect have already been approved by the United States Environmental Protection Agency (EPA)^1^.

The proposed RNAi insecticide, developed by Monsanto and Dow Agrosciences, will be known as SmartStax Pro^®^. This plant-incorporated protectant (PIP) will employ a pyramid strategy: several different Bt-proteins, as well as dsRNA targeting the WCR *Snf7* gene, will be expressed in this plant (Head et al., 2017). Bt-proteins, also known as crystalline toxins, insert themselves into the gut epithelium of the insect, causing gut paralysis and resulting in the death of the insect (Copping and Menn, 2000). On the other hand, downregulation of Snf7, a gene that plays an essential role in protein trafficking, will also result in mortality (Bolognesi et al., 2012). This combined strategy is designed to lead to the swift death of the insect, while also reducing the chances that insects will develop resistance against this PIP (Head et al., 2017). As RNAi is a budding technology within the field of agriculture, it is likely only a matter of time before SmartStax Pro^®^ and other, yet to be discovered insecticidal strategies, will appear on the market.

## Insect Viral Infections and RNAi-Based Antiviral Immunity

Insects represent the largest group of animals on Earth in terms of biodiversity, with an estimated number of 5.5 million different species (Stork, 2018). This diversity reflects in a matching range of infecting viruses, which in addition to positively or negatively affecting insect populations, can also have a major impact on human well-being (Miller and Ball, 1998; Roossinck, 2011). In this section, important concepts regarding the patterns of viral infection pathogenesis will be addressed. Then, relevant insect disease-causing and persistent viral infections will be reviewed. At last, the use of viruses for insect biological control, as well as the potential use of the RNAi technology to protect beneficial insects from harmful viral infections will be discussed.

### Patterns of Viral Infection – From Lethality to Non-pathogenicity

Viral infections can be classified according to their effect on the host, ranging from presenting no obvious harmful symptoms to being highly pathogenic or even lethal. These distinct outcomes exist in a variable range and are generally linked to different levels of viral particle production. Therefore, although this classification is not established beyond doubt, efforts have been made to classify them in three main groups, namely: acute, persistent and latent. Acute infections are characterized by high levels of viral replication and increased viral particle production. Generally, these infections are limited in time; either by the death of the host or by the clearance of the virus by the host immune system. On the other hand, persistent infections are characterized by constant, but relatively low, levels of viral replication and of viral particle production. These infections can manifest themselves for longer periods of time as often an equilibrium is established between the attack and counterattack strategies of the virus-host system. Although some persistent infections have the potential to cause variable levels of pathogenic effects, clear effects on fitness are often not observed. Finally, latent infections consist in the presence of the viral genome in the host cell without actual production of viral particles. The viral genome (in DNA form) can remain latent either as an episome or can be integrated in the host genome as a provirus. During this latency, viruses maintain the potential to resume viral replication and start producing viral particles, a process which is referred to as reactivation (Boldogh et al., 1996; Swevers et al., 2013; Nathanson and González-Scarano, 2016).

**FIGURE 2:**
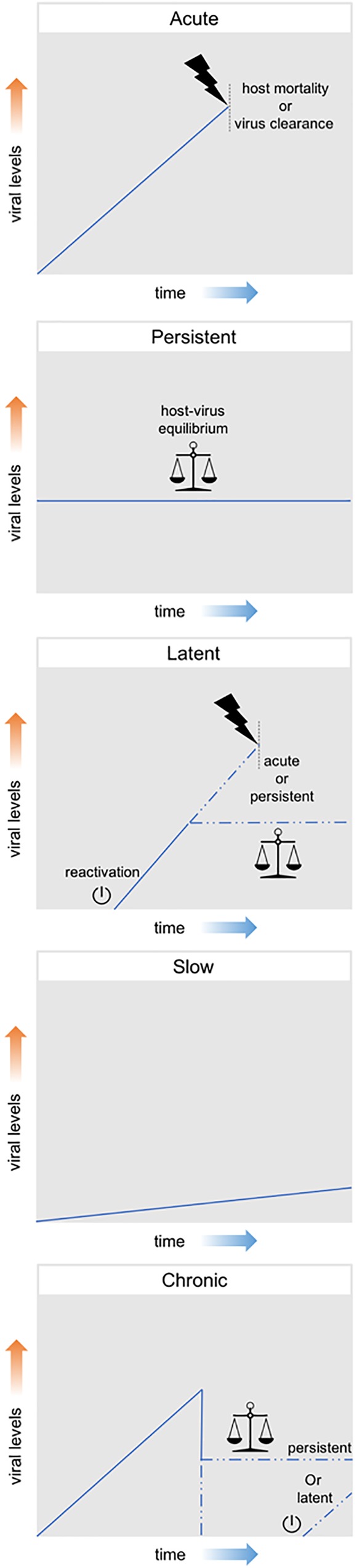
Patterns of viral infection. Acute infections are represented by a high increase in viral levels and are limited in time either by the death of the host or by the clearance of the virus by the host immune system. Persistent infections consist in constant, but relatively low, viral levels and can manifest themselves for long periods of time. Latent infections consist in the presence of the viral genome in the host cell without actual production of viral particles. During this latency, viruses maintain the potential to resume viral replication and start producing viral particles (reactivation). Chronic infections are generally defined as the outcome of an acute infection in which neither host mortality nor virus clearance occur, meaning a persistent or latent outcome derived from an acute infection. Slow infections are characterized by a slow, but not constant, increasing in viral levels overtime.

Additionally, the terms chronic and slow infection are often used, although mostly in the context of human viral diseases. A chronic infection is generally defined as the outcome of an acute infection in which neither host mortality nor virus clearance occur, meaning a persistent or latent outcome derived from an acute infection. In a slow infection, viral replication and particle production are slow but not constant, increasing overtime (Boldogh et al., 1996; Virgin et al., 2009). Figure 2 summarizes these different patterns of viral infection.

### Acute Viral Infections in Insects – Disease in Beneficials and Control Strategies for Pests

Clear examples of acute viral infections are the ones affecting beneficial insects, such as bees and economically important lepidopteran species. Recently, worrying losses in bee populations have been observed. These are typically associated with environmental pollution, specific pesticides or the presence of parasitic infections (Goulson et al., 2015). However, the impact of diseases caused by different viruses, though often overlooked, cannot be ignored. In fact, several studies have identified multiple harmful viruses infecting honeybees and bumblebees (Bailey et al., 1963, 1964, 1982; Bailey, 1969; Bailey and Woods, 1974, 1977; Benjeddou et al., 2001; Maori et al., 2007; Rana et al., 2011; Granberg et al., 2013; Chen et al., 2014; Meeus et al., 2014; Roberts and Anderson, 2014; Ravoet et al., 2015; Ueira-Vieira et al., 2015; Benaets et al., 2017; Natsopoulou et al., 2017). In addition, in the specific case of honeybees, losses have also been linked to the so-called Colony Collapse Disorder (CCD) which, in general terms, results in the sudden death of colonies. Although the specific causes of this phenomenon are still to be unraveled, it is thought that the aforementioned factors have been correlated with it, including the prevalence of several viral diseases (Brutscher and Flenniken, 2015). Another relevant example can be found in silkworms, whose viral diseases often cause sizeable economic losses to the sericulture industry in Asiatic countries (Sanakal et al., 1996; Jiang et al., 2016; Gani et al., 2017; Chen et al., 2018). Table 2 presents an overview of viruses with significant impact on bees and silkworms. In addition, viruses affecting economically important shrimps are also contemplated.

**Table 2:** Overview of viruses with a significant impact on beneficial insects and on economically relevant shrimps.

Beneficial arthropods	Infecting virus	Genome - Taxonomy (Genus)	Host stage	Symptoms acute stage	Reference
**Honey bee**					
	Sacbrood Virus	+ssRNA – Iflavirus	Pupae/Adults	Failure to pupate, death	Gisder and Genersch, 2017
	Varroa destructor virus-1	+ssRNA – Iflavirus	Pupae/Adults	Deformed wings, shortened lifespan, Colony collapse	Natsopoulou et al., 2017
	Chronic Bee Paralysis Virus	+ssRNA – Noda/Tombus-virus	Adults	Paralysis, death	Bailey et al., 1963
**Honey bee and Bumblebee**					
	Black Queen Cell Virus	+ssRNA – Cripavirus	Pre-/Pupae	Decomposed and black pre-/pupae	Bailey and Woods, 1977
	Kashmir Bee Virus	+ssRNA – Dicistrovirus	Adults/pupae	Reduced fecundity, death	Bailey and Woods, 1977; Meeus et al., 2014
	Israeli Acute Paralysis Virus	+ssRNA – Dicistrovirus	Adults	Reduced fecundity, paralysis, darkening, hair loss, death	Chen et al., 2014; Meeus et al., 2014
	Acute Bee Paralysis Virus	+ssRNA – Dicistrovirus	Adults	Paralysis, darkening, hair loss, death	Bailey et al., 1963; de Miranda et al., 2010
	Deformed Wing Virus	+ssRNA – Iflavirus	Pupae/Adults	Deformed wings, shortened lifespan, Colony collapse	Benaets et al., 2017
	Slow Bee Paralysis Virus	+ssRNA – Iflavirus	Pupae/Adults	Paralysis of anterior legs, death	Bailey and Woods, 1974; Niu et al., 2016
**Silkworm**					
	*Bombyx mori* Nucleopolyhedrosis virus	dsDNA – Alphabaculovirus	Larvae/Pupae/Adults	Molting failure, hyperactivity, translucent skin, white hemolymph, death	Steinhaus, 1949
	*Bombyx mori* Cypovirus	dsRNA – Cypovirus	Larvae/Pupae/Adults	Delayed larval growth, failure to pupate	Payne and Rivers, 1976
	Infectious Flacherie Virus	+ssRNA – Iflavirus	Larvae	Flaccidity, retarded growth, death	Himeno et al., 1974; Shimizu, 1975
	*Bombyx mori* Densovirus	ssDNA – Iteravirus	Larvae	Flaccidity, retarded growth, death	Shimizu, 1975
**Penaeid shrimp**					
	Taura Syndrome Virus	+ssRNA – Cripavirus	All stages	Lethargy, epithelial necrosis of entire body, death	Hasson et al., 1995
	Infectious Hypodermal and Hematopoietic Necrosis Virus	ssDNA – Brevidensovirus	Juvenile, Adults	Cuticular deformities, impaired growth, death	Kalagayan et al., 1991; Shike et al., 2000; Prasad et al., 2017
	Yellow Head Virus	+ssRNA – Okavirus	All stages	Yellow discoloration, systemic necrosis, death	Sittidilokratna et al., 2008
	White Spot Syndrome Virus	dsDNA – Whispovirus	Juveniles, Adults	White spots, red/yellow discoloration, death	Sudha et al., 1998; Escobedo-Bonilla et al., 2008
	Infectious Myonecrosis Virus	dsRNA – Unclassified	Juveniles, Subadults	White spots in, and necrosis of, skeletal muscles, death	Poulos et al., 2006; Prasad et al., 2017

In addition to the viruses that cause harmful infections in beneficial insects, others can use insects as vectors to infect other animals or plants – arthropod-borne viruses, or arboviruses. Important examples are the ones responsible for human diseases, transmitted via mosquitoes (e.g., Chikungunya Virus, Dengue Virus, Yellow Fever Virus, West Nile virus, Japanese Encephalitis Virus and Zika Virus) (Ng et al., 2011; Shi et al., 2015; Minkeu and Vernick, 2018); and plant viruses which may be deleterious to crops (Ng and Perry, 2004; Hohn, 2007; Whitfield et al., 2015).

Insect viruses can also have a positive impact on human wellbeing. This is the case for viruses whose hosts are considered pest species. Because of this, insect viruses have long been researched for use as pest control agents. Some examples of viruses as delivery systems for RNAi were already discussed. Until recently, the use of microbial bio-insecticides has remained stagnant. However, increased concern about health and pollution hazards, environmental awareness, increased governmental restrictions on synthetic pesticides, advances in farming technology and other factors are fueling a solid growth in the biopesticide market. To be applicable as biopesticides, the viruses must comply with several requirements: they must be specific, highly virulent, and lethal to the targeted pest insect, while preferably also inducing epizootics (Fuxa, 1991). Despite the large diversity in entomopathogenic viruses, commercial research and use is mainly restricted to the family of *Baculoviridae*. Even though baculoviruses can infect arthropods belonging to several insect orders, only Lepidoptera-specific viruses belonging to the genera Nucleopolyhedrosis virus (Alphabaculovirus) and Granulovirus (Betabaculovirus) have been developed into commercial products (Lacey et al., 2015). The widescale use of Baculoviruses can be explained by the already extant knowledge and expertise on this family of viruses as well as their useful characteristics. Baculoviruses display remarkable specificity and infections are highly lethal. During the late stage of infection, Baculoviruses produce occlusion bodies (OBs). These OBs increase resistance to the environment and make baculoviral insecticides easier to store and apply in the field. The major drawbacks of viral biopesticides are: the current absence of practical mass production systems resulting in high production costs; the (relatively) slow kill rate; short shelf-life and inconsistent field performance (Lacey et al., 2015; Arthurs and Dara, 2018).

To date, the most widely used viral biopesticide is the *H. armigera* NPV, with over 10 manufacturers in China alone and new product registrations occurring on a yearly basis^2^. Other important viral agents are the *S. exigua* NPV, *S. litura* NPV and *Cydia pomonella* GV. A comprehensive list of virus-based commercial insecticide products has been assembled by Lacey et al. (2015) and Arthurs and Dara (2018).

### Virome and Insect Persistent Viral Infections

The idea that persistent viral infections are ubiquitous has recently started to emerge. In fact, the word ‘virome’ is often used nowadays and this field of research has gained a lot of interest in insects. This is due to three main reasons: (1) the advent of genomic and transcriptomic techniques; (2) the growing idea that the microbiome, including the virome, has the potential to interfere (both positively and negatively) in many biological processes; (3) and the establishment of viruses as crucial drivers of evolution. The latter has gained interest not only due to the ability of genetic mobile elements to cause mutagenesis, but also due to their potential capacity of providing hosts with beneficial gene-regulatory machinery (Swevers et al., 2013; Massart et al., 2014; Bikel et al., 2015; Koonin, 2016; Chuong et al., 2017; Nouri et al., 2018).

In addition to the major direct impact of insect viruses on human life, as reviewed above; insect viral infections might play crucial roles on the ecological equilibrium of our planet. Therefore, it is of great interest to understand the mechanisms underlying the establishment and maintenance of insect viromes. In this context, persistent viral infections gain special relevance. Since these do not always cause obvious pathogenesis, their existence is often neglected. However, recently, identification of persistent viruses in insects has become recurring, with several reported cases both *in vivo* and in cultured cells (Katsuma et al., 2005; Habayeb et al., 2006; Li et al., 2007; Wu et al., 2010; Jovel and Schneemann, 2011; Iwanaga et al., 2012; Ma et al., 2014; Suzuki et al., 2015; Swevers et al., 2016; Santos et al., 2018). Interestingly, whether an infection is persistent or acute does not depend only on the virus itself, but also on the host. As demonstrated by several loss-of-function and deep sequencing studies, the role of RNAi in the established equilibrium between the persistent virus and the insect host is clear (Wu et al., 2010; Goic et al., 2013; Zografidis et al., 2015; Petit et al., 2016; Santos et al., 2018). However, the possible role of still unidentified factors has to be considered. A particularly interesting example is the Flock House Virus (FHV), which is known to cause persistent infections in lepidopteran cell lines and acute infections in crickets and flies (Longdon et al., 2012; Swevers et al., 2016). Remarkably, and by still unknown mechanisms, FHV is able to cause the two types of infection in *D. melanogaster* S2 cells (Goic et al., 2013). Further research regarding the diversity of insect viromes and the (RNAi-based) mechanisms involved in persistent-to-acute viral-host interactions would be of great value to understand their influence on several physiological processes; as well as their potential to contribute to efficient strategies to protect beneficial insects from harmful pathogens and to control dangerous pest insects.

### RNAi-Based Antiviral Immunity to Protect Beneficial Insects

As discussed in the previous sections, it is clear that a deep understanding of the interactions between insects and their viruses is of great value. In addition, the current demand to control insect viral infections stresses the need to search for original approaches to fight these infections. Since RNAi is the main insect antiviral immune response, it is only logical to think of this mechanism as a potential form to protect beneficial insects against harmful viral infections.

In this context, the use of virus specific dsRNA aiming to trigger the RNAi pathway against viral infections has already been explored in bees. More specifically, delivery of targeted virus dsRNA by injection or feeding has been demonstrated to be effective in protecting honeybees against several relevant viral infections (Maori et al., 2009; Hunter et al., 2010; Liu et al., 2010; Desai et al., 2012; Flenniken and Andino, 2013; Brutscher et al., 2017). In line with these findings, feeding virus-specific dsRNA to bumblebees has been demonstrated to act against the IAPV infection (Piot et al., 2015). Surprisingly, delivery of non-specific dsRNA has revealed to trigger an antiviral response in both honeybees and bumblebees (Flenniken and Andino, 2013; Piot et al., 2015; Brutscher et al., 2017).

Similar approaches have been investigated in lepidopteran insects with promising results. First, transfection or expression of virus-specific dsRNA in cell lines was shown to result in lower viral levels (Valdes et al., 2003; Isobe et al., 2004; Kanginakudru et al., 2007). Then, transgenic *B. mori* silkworms expressing virus-specific dsRNA have been reported to exhibit higher survival rates upon NPV infection on several occasions. In fact, this approach has been demonstrated to be effective in a commercially valuable silkworm strain (Isobe et al., 2004; Kanginakudru et al., 2007; Subbaiah et al., 2013). In addition, a similar strategy has been successfully tested to obtain protection of the silkworm to the *B. mori* cytoplasmic polyhedrosis virus (BmCPV) (Jiang et al., 2017). At last, injection of virus-specific dsRNA has been demonstrated to protect the mealworm beetle, *Tenebrio molitor*, against viral infection as well (Valdes et al., 2003). Notably, comparable approaches have also been successfully applied in two economically relevant crustacean species (Robalino et al., 2004, 2005; Tirasophon et al., 2005; Yodmuang et al., 2006; Attasart et al., 2010; Labreuche et al., 2010; Bartholomay et al., 2012). Recently, *Trichoplusia ni* High Five cells overexpressing key components of the RNAi machinery, namely *B. mori* Dicer2 and Argonaute2, have been reported to present reduced CrPV-induced mortality (Santos et al., 2018). This tactic remains to be tested *in vivo* and with regard to infections by other viruses. However, since RNAi is a broadly-acting antiviral immune mechanism in insects and considering that improvement of the RNAi response is observed in transgenic *B. mori* larvae overexpressing Argonaute2 (Li Z. et al., 2015), this constitutes a promising approach. Table 3 presents a summary of the investigated strategies to obtain improved antiviral defense in insects, as well as in economically relevant crustacean species.

**Table 3:** Summary of the investigated strategies to obtain improved antiviral defense in insects and in economically relevant crustacean species.

Species	*In vivo*/*in vitro*	Virus	Strategy	Outcome	Reference
*A. mellifera*, the western honeybee	*In vivo*	IAPV	Oral delivery of virus-specific dsRNA	Lower mortality; lower viral transcript levels	Maori et al., 2009
*A. mellifera*, the western honeybee	*In vivo* - colonies	IAPV	Oral delivery of Remebee-I (a IAPV-specific dsRNA product)	Florida colony: higher bee population per hive; higher adult forager activity; higher hive total weight gain (honey); Pennsylvania colony: higher hive total weight gain (honey); lower Nosema levels.	Hunter et al., 2010
*A. mellifera*, the western honeybee	*In vivo*	DWV	Oral delivery of virus-specific dsRNA	Lower proportion of adult bees with deformed wings; lower viral transcript levels; adult survival was not affected	Desai et al., 2012
*A. mellifera*, the western honeybee	*In vivo*	SINV-GFP	Injection of virus-specific and unspecific dsRNA	Lower viral abundance	Flenniken and Andino, 2013
*A. mellifera*, the western honeybee	*In vivo*	SINV-GFP	Injection of virus-specific and unspecific dsRNA	Lower viral abundance	Brutscher et al., 2017
*A. cerana*, the eastern honeybee	*In vivo*	CSBV	Oral delivery of virus-specific dsRNA	Lower larvae mortality; lower viral transcript levels	Liu et al., 2010
*A. Cerana*, the eastern honeybee	*In vivo*	CSBV	Oral delivery of virus-specific dsRNA	Lower larvae mortality; lower viral transcript levels	Zhang et al., 2016
*B. terrestris, the bumblebee*	*In vivo*	IAPV	Oral delivery of virus-specific and unspecific dsRNA	Lower viral transcript levels in the head	Piot et al., 2015
*Tenebrio molitor*, the mealworm beetle	*In vivo*	AcNPV-GFP	Injection of virus-specific dsRNA	Lower mortality	Valdes et al., 2003
*Spodoptera frugiperda*, the fall armyworm	*In vitro* – Sf21 cells	AcNPV-GFP	Transfection with virus-specific dsRNA	Reduced fluorescence; lower viral protein levels; lower viral transcript levels; lower amount of viral particles; reduced cell morphological changes	Valdes et al., 2003
*B. mori*, the silkworm	*In vitro* – BmN cells	BmNPV	Expression (transient transfection) of virus-specific dsRNA	Lower virus titer in the cell culture medium	Isobe et al., 2004
*B. mori*, the silkworm	*In vitro* – BmN cells	BmNPV	Expression (constitutive transfection) of virus-specific dsRNA	Lower virus titer in the cell culture medium; lower viral transcript levels in the cells	Isobe et al., 2004
*B. mori*, the silkworm	*In vivo*	BmNPV	Transgenic animals expressing virus-specific dsRNA	Reduced levels of viral DNA in the hemolymph	Isobe et al., 2004
*Spodoptera frugiperda*, the fall armyworm	*In vitro* – Sf9 cells	AcNPV	Expression (constitutive transfection) of virus-specific dsRNA	Lower virus titer in the cell culture medium; reduced OBs in the cells	Kanginakudru et al., 2007
*B. mori*, the silkworm	*In vivo*	BmNPV and GFP-BmNPV	Transgenic animals expressing virus-specific dsRNA	Lower mortality; reduced levels of OBs in the hemolymph; reduced fluorescence; reduced viral protein levels; lower viral transcript levels	Kanginakudru et al., 2007
*B. mori*, the silkworm	*In vivo*	BmNPV	Transgenic animals expressing single or multiple virus-specific dsRNAs (targeting different genes)	Lower mortality rates; lower levels of OBs in the hemolymph; lower viral DNA levels; reduced viral protein levels	Subbaiah et al., 2013
*B. mori*, the silkworm	*In vivo*	BmCPV	Transgenic animals expressing single or multiple virus-specific dsRNAs (targeting different genes)	Lower mortality rates; lower viral transcript levels	Jiang et al., 2017
*T. ni*, the cabbage looper	*In vitro* – High five cells	CrPV	Expression (transient transfection) of BmDicer2 and BmArgonaute2	Reduced cell mortality	Santos et al., 2018
*Litopenaeus vannamei*, the pacific white leg shrimp	*In vivo*	TSV and WSSV	Injection of unspecific dsRNA	Lower mortality; lower accumulation of viral particles; lower levels of tissue damage.	Robalino et al., 2004
*Litopenaeus vannamei*, the pacific white leg shrimp	*In vivo*	WSSV	Injection of virus-specific dsRNA	Lower mortality	Robalino et al., 2005
*Litopenaeus vannamei*, the pacific white leg shrimp	*In vivo*	WSSV	Injection of unspecific dsRNA of multiple sizes (50–200 bp)	Lower mortality	Labreuche et al., 2010
*Litopenaeus vannamei*, the pacific white leg shrimp	*In vivo*	WSSV	Injection of virus-specific and unspecific dsRNA	Lower mortality	Bartholomay et al., 2012
*Penaeus monodon*, the Asian tiger shrimp	*In vitro* – Oka cells	YHV	Transfection of virus-specific and unspecific dsRNA	Reduced cytopathic effects; lower viral transcript levels in the cell medium; lower viral protein levels	Tirasophon et al., 2005
*Penaeus monodon*, the Asian tiger shrimp	*In vivo*	YHV	Injection of virus-specific dsRNA	Lower viral transcript levels; reduced mortality	Yodmuang et al., 2006
*Penaeus monodon*, the Asian tiger shrimp	*In vivo*	YHV	Injection of unspecific dsRNA	Lower mortality	Yodmuang et al., 2006
*Penaeus monodon*, the Asian tiger shrimp	*In vivo*	DNV	Injection of virus-specific dsRNA	Lower viral DNA levels	Attasart et al., 2010

*IAPV, Israel Acute Paralysis Virus; DWV, Deformed Wing Virus; SINV, Sindbis Virus; CSBV, Chinese Sacbrood Virus; NPV, Nuclear Polyhedrosis Virus; CPV, Cytoplasmic Polyhedrosis Virus; CrPV, Cricket Paralysis Virus; TSV, Taura Syndrome Virus; WSSV, White Spot Syndrome Virus; YHV, Yellow Head Virus; DNV, Densovirus; GFP, Green Fluorescence Protein; OBs, Occlusion Bodies; Ac, Autographa californica; Bm, B. mori.*

Considering these potential strategies to control insect viral diseases, the use of genetically engineered insects deserves special attention due to the risks of environmental contamination. In fact, up to date, the release of transgenic insects has been limited to sterile animals, with the aim of reducing pest species populations (Reeves and Phillipson, 2017; Wilke et al., 2018). However, the use of genetically engineered beneficial insects, as would be the eventual goal for various species of bees, would mostly require the maintenance of viable populations and therefore the use of fertile transgenic animals. On the other hand, the breeding of domestic silkworms closely depends on humans and is, therefore, highly controlled. Thus, the use of transgenic moths in sericulture is more feasible and might hold great potential with lower risks compared to the creation of other valuable transgenic species. In this context, it is important to keep in mind that several challenges will need to be overcome before such innovative strains can be obtained. For example, special regard should be payed to the productivity and fitness of such transgenic lines, as well as to the maintenance of the phenotype throughout several generations (Jiang and Xia, 2014). Furthermore, a last consideration should be given to the eventual development of resistance by these viruses. Thus, before actual implementation, approaches to minimize this issue should be contemplated, such as the use of inducible expression systems which would be activated only in the case of viral disease, or the alternate expression of different transgenes.

## Conclusion and Future Prospects

**FIGURE 3:**
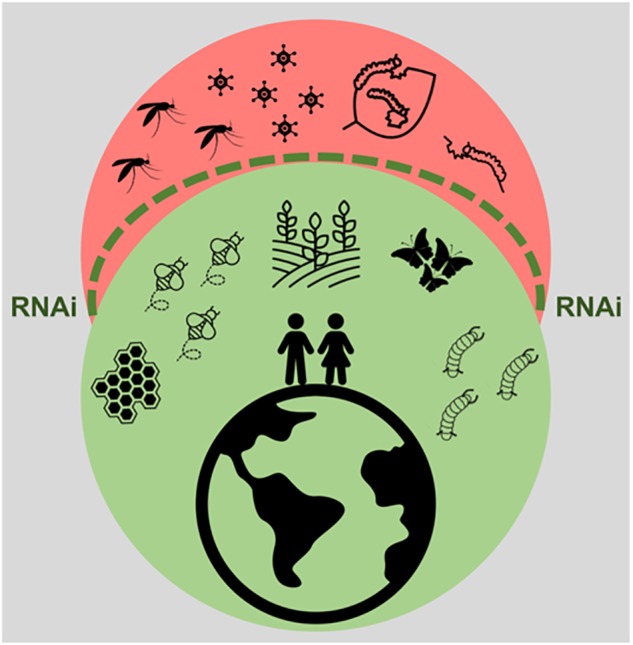
Impact of RNAi technology and of insect viral infections in human life. The RNAi technology, represented by the dashed green line, promises to exert protection against pest insects, such as the ones threatening crop production and the ones constituting vectors for viral diseases. This technology also holds potential to protect beneficial insects from harmful viral infections. In addition, insect viruses constitute important bio-insecticides.

It is clear that the impact of RNAi technology and of insect viral infections on human life cannot be underestimated. In fact, in agricultural and industrial contexts, this is likely to become even more prominent in the years to come. In the first place, RNAi technology poses great potential to contribute to highly specific insect control strategies through delivery of dsRNA to pest species. Although the application of this technique has so far been limited by the variable RNAi efficiency amongst economically important insects, delivery systems provide a promising solution. Of these, an incredible wealth of options is available, with encouraging success rates. In the second place, insect baculoviruses form an interesting class of highly species-specific insecticides, which are currently under use. Due to the increasing knowledge on insect virus diversity, the potential use of other viral families cannot be excluded. In addition, the RNAi mechanism shows great potential as a combatant against viruses, which form an undeniable threat to beneficial insects. The efficient delivery of virus-specific dsRNA is a promising approach to protect beneficial insects such as pollinators. In addition, in the case of silkworms, the use of transgenic lines, resistant against such viral infections seems possible in the foreseeable future. This paper reviews the current literature on practical applications based on insect viruses and RNAi, as summarized in Figure 3. Although some of the described aspects still need to be thoroughly researched and therefore have to be considered with caution, this is an undeniably exciting field of research, full of potential.

## Author Contributions

EV, DS, LM, T-WV, and JB conceived the manuscript. EV, DS, LM, and T-WV wrote the parts of the text. EV and DS worked on the structure and prepared the final version of the manuscript. EV, DS, and LM prepared the figures. EV, DS, and T-WV prepared the tables. JB corrected the manuscript and suggested further improvements.

## Conflict of Interest Statement

The authors declare that the research was conducted in the absence of any commercial or financial relationships that could be construed as a potential conflict of interest.

## Footnotes

1 https://www.epa.gov/newsreleases/epa-registers-innovative-tool-control-corn-rootworm

2 https://agrow.agribusinessintelligence.informa.com/AG012300/Biopesticide-development-in-China
